# Development and validation of a risk prediction model for feeding intolerance in neurocritical patients with enteral nutrition

**DOI:** 10.3389/fnut.2024.1481279

**Published:** 2024-10-23

**Authors:** Rong Yuan, Lei Liu, Jiao Mi, Xue Li, Fang Yang, Shifang Mao

**Affiliations:** ^1^Neurological Intensive Care Unit, Deyang People's Hospital, Deyang, China; ^2^Department of Nursing, Deyang People's Hospital, Deyang, China; ^3^Department of Nursing, Affiliated Hospital of Southwest Medical University, Luzhou, China

**Keywords:** neurocritical, feeding intolerance, risk factors, prediction model, enteral nutrition

## Abstract

**Background:**

This study collects and analyzes clinical data on enteral nutrition therapy in neurocritical patients, develops and validates a feeding intolerance (FI) risk prediction model, and provides a theoretical basis for screening patients with high risk of feeding intolerance (FI) and delivering personalized care.

**Methods:**

A convenience sampling method was employed to select 300 patients who were admitted to a tertiary hospital in China for early enteral nutrition therapy in the neurointensive care unit between April 2022 and December 2022. Independent risk factors for FI were identified using univariate and logistic regression analyses. A prediction model was established, and the goodness of fit and discriminant validity of the model were evaluated.

**Results:**

The incidence of FI in neurocritical patients receiving enteral nutrition was 71%. Logistic regression analysis identified age, Glasgow Coma Scale (GCS) scores, Acute Physiology and Chronic Health Evaluation II (APACHE II) scores, mechanical ventilation, feeding via the nasogastric tube route, hyperglycemia, and low serum albumin as independent risk factors for the development of FI (*p* < 0.05). The predictive formula for FI risk was established as follows: Logit *p* = −14.737 + 1.184 × mechanical ventilation +2.309 × feeding route +1.650 × age + 1.336 × GCS tertile (6–8 points) + 1.696 × GCS tertile (3–5 points) + 1.753 × APACHE II score + 1.683 × blood glucose value +1.954 × serum albumin concentration. The Hosmer–Lemeshow test showed *χ^2^* = 9.622, *p* = 0.293, and the area under the ROC curve was 0.941 (95% confidence interval: 0.912–0.970, *p* < 0.001). The optimal critical value was 0.767, with a sensitivity of 85.9%, a specificity of 90.8%, and a Youden index of 0.715.

**Conclusion:**

The early enteral nutrition FI risk prediction model developed in this study demonstrated good predictive ability. This model can serve as a valuable reference for effectively assessing the risk of FI in neurocritical patients, thereby enhancing clinical outcomes.

## 1 Introduction

Enteral nutrition is the preferred method of providing nutrition to critically ill patients. Both the Society of Critical Care Medicine and the American Society for Parenteral and Enteral Nutrition recommend that enteral nutrition should be initiated as early as possible within 24–48 h of admission to the care unit when there are no contraindications to enteral nutrition (1). Early enteral nutrition helps nourish the gastrointestinal mucosa and enhance neuroendocrine function, thereby protecting the intestinal mucosal barrier and immune function (2–5). Enteral nutrition has been found to be associated with reduced infection rates, accelerated wound healing, reduced duration of invasive mechanical ventilation, shorter length of stay in the care unit, and reduced mortality rates (6, 7). However, during clinical practice, acute gastrointestinal dysfunction often occurs in critically ill patients under intense stress, with feeding intolerance (FI) being the most common clinical manifestation (8). According to some studies, the incidence of FI in critically ill patients ranges from 30.5 to 75% (9–12).

The neurointensive care unit (NCU) is a specific facility that mainly admits and treats critically ill patients with neurological diseases and concurrent or potential organ dysfunction, such as severe ischemic stroke, cerebral hemorrhage, severe traumatic brain injury (TBI), and increased intracranial pressure, among others (13). These patients often experience a higher incidence of FI due to impaired consciousness, abnormal brain-gut axis regulation, vagal inhibition, and so on (14, 15). The occurrence of FI in NCU patients poses a substantial risk for poor prognosis, including increased duration of invasive mechanical ventilation, prolonged hospitalization, increased infection rate, and mortality rate, among others (16–18).

However, the awareness level of FI among healthcare professionals may vary due to the absence of a standardized definition. In a 2014 meta-analysis (19), up to 43 clinical manifestations of FI were observed, such as vomiting, regurgitation, diarrhea, constipation, increased abdominal girth, subjective discomfort, and aspiration. The commonly used definition of FI in clinical practice is based on the 2012 European Society of Critical Care Medicine definition of FI (20), which includes the following three aspects: the presence of gastrointestinal discomfort symptoms, such as vomiting, abdominal distension, diarrhea, constipation, loss of bowel sounds, and a high gastric residual volume (GRV) >500 mL/d; the discontinuation of enteral nutrition due to gastrointestinal bleeding or other factors; and the failure to achieve an energy intake of 20 kcal/kg/d within 72 h of the start of enteral nutrition. FI is considered to have occurred if one or more of the above criteria are met.

Current preventive measures for FI mainly include early identification of risk factors and early intervention. Numerous factors for FI have been identified, including mechanical ventilation (19), hyperglycemia (8), and hypoproteinemia (21). For early detection, methods such as ultrasound (22, 23), intra-abdominal pressure monitoring (24), and biomarkers (25) can be employed; however, their specificity remains to be tested. In addition, although some studies have proposed risk factors for FI and established risk prediction models, the majority of these studies target critically ill patients or patients with severe pancreatitis and fewer for NCU patients. Therefore, this study aims to identify the risk factors and develop a prediction model for FI during early EN in NCU patients in order to recognize the high-risk group at an early stage, thereby providing a valuable clinical reference for timely intervention and improvement of patient outcomes.

## 2 Materials and methods

### 2.1 Study design

This prospective cohort study was conducted in a 24-bed NCU at a tertiary general hospital in China, which also serves as a teaching hospital. The study took place between April 2022 and December 2022. No additional interventions were conducted other than the necessary assessments. Therefore, data collection was not burdensome for patients, and data were collected and analyzed anonymously. The study was also approved by the hospital’s Ethics Committee under the ethical code 2022-04-042-K01.

### 2.2 Enrollment

A convenience sampling method was adopted to collect clinical data on 300 patients admitted to the NCU for early enteral nutrition. The inclusion criteria for this study are as follows: (1) critically ill patients with neurological disease and existing or potential organ dysfunction; (2) aged ≥18 years; (3) first placement of nasogastric/nasoenteric tubes; (4) enteral feeding was initiated within 48 h of admission; (5) nasal feeding for ≥7 days; and (6) patients/family members signed an informed consent form and were willing to cooperate with the investigator.

The exclusion criteria are as follows: (1) severe nutritional disorders, digestive insufficiency, and cirrhosis; (2) previous intestinal obstruction; (3) bleeding from the esophagus, stomach, or intestines; and (4) incomplete data.

### 2.3 Definition of feeding intolerance

According to the FI definition of the European Society of Critical Care Medicine (20) and considering the disease characteristics of NCU patients, the judgment criteria for FI in this study were as follows: ① Vomiting/reflux: It refers to the passage of gastric contents through the esophagus and out of the body through the mouth. The criterion for judging vomiting/reflux was the presence of nutrient fluid in the mouth or spillage of nutrient fluid from a nasogastric or nasoenteric tube when the tube is opened. ② Diarrhea: Patients with >3 bowel movements per day, feces with a water content of 80% or more, and unformed consistency were considered to have diarrhea. Using the HART diarrhea scoring method (26), each bowel movement was scored, and diarrhea was considered to be present if the 24-h cumulative total score was ≥12. ③ Constipation: This is defined as no bowel movement for 3 consecutive days or one bowel movement in 2–3 days, with dry and hard stool is dry and hard amounting to <50 g. ④ Aspiration: This refers to the aspiration of gastric contents via the airway. ⑤ High gastric residual volume (GRV): A volume of ≥500 mL per day was considered high; ⑥ Gastrointestinal Bleeding: This is determined after the patient vomited, regurgitated fluid, or had visible bloody fluid in the stool along with a positive occult blood test and/or after the diagnosis has been confirmed by a physician, such as through gastroscopy.

### 2.4 Method of nutritional support

Nurses in NCU followed the doctor’s orders to initiate enteral nutrition therapy for patients. The nursing team in the department developed an enteral nutrition therapy program, drawing upon several key resources for guidance. These resources included “Expert Consensus on Enteral Feeding Nursing for Patients with Severe Neurocritical Diseases (2022 Edition)” (27), “Nursing Practice Guideline For Enteral Nutrition In Patients With Stroke (2021 Edition)” (28), and the group standard “Intubation And Maintenance Of Nasointestinal Tube In Adult Patients” (29) issued by the Chinese Nursing Association in January 2022. The program includes several practices as follows: (1) utilizing a special pump to administer the EN solution at a temperature of approximately 37–40°C; (2) placing special signs for EN beside the patient’s bed to distinguish it from intravenous infusion; (3) flushing the tubes with 20–30 mL of warm water before and after each feeding, as well as every 4 h during the infusion of EN. Every shift, the exposed portion of the feeding tube should be inspected, and the nasal cavity should be checked. Moreover, the airbag pressure should be maintained at 25–30 cmH2O for patients with artificial airways, and subglottic suction should be performed simultaneously.

### 2.5 Candidate predictors

After analyzing the relevant literature on FI, integrating this information with clinical data, and engaging in discussions with experts, we identified risk factors that may affect the occurrence of FI in neurocritical patients. We identified 27 potential predictors categorized into four main groups: (1) individual patient factors: age, sex, body mass index (BMI) (30), smoking history, drinking history; (2) disease characteristics: primary diagnosis, past history, Glasgow Coma Scale (GCS) scores (31), nutritional risk screening (NRS) 2002 scores (32), Acute Physiology and Chronic Health Evaluation II (APACHE II) scores (33); (3) therapeutic factors: whether surgery was performed, use of mechanical ventilation, target temperature management, use of two or more antibiotics, use of vasoactive drugs, sedatives, analgesics, potassium preparations, acid inhibitors, feeding routes, and nasal feeding preparations; (4) monitoring data: body temperature, level of blood glucose, serum albumin, prealbumin, serum lactate, and potassium.

### 2.6 Data collection

The data for this study were obtained from electronic medical records and critical care information systems. Data collection for this study began when enteral nutrition was initiated in NCU patients. The collector conducted a 7-day data collection of patients’ FI at 03:00, 09:00, 15:00, and 21:00 daily. Two investigators collected the data with mutual verification of completeness, authenticity, and accuracy. Additionally, a dedicated individual was tasked with maintaining complete data records.

### 2.7 Sample size

According to the sample size calculation criteria based on the logistic regression analysis proposed by Gao and Zhang (34), the sample size should be 5–10 times the number of independent variables divided by the incidence of disease. A total of 19 independent variables were included in this study. With the incidence of FI at approximately 68.3%, as determined by a small pre-survey in the hospital, and assuming a 10% loss to follow-up, the minimum sample size required for this study is calculated as follows: 27*5*(1 + 10%)/68.3% = 217.

### 2.8 Statistical analysis

Patients were divided into FI and non-FI groups based on the diagnostic criteria. Excel 2013 software was used to create a double-entry database for double entry for data validation, and SPSS Statistics version 25.0 software was used for data analysis. Count data were expressed as frequency and percentage (%), and the chi-Squared (χ^2^) test was performed between the two groups. Normal measure data were expressed as mean ± standard deviation (SD), and two independent samples *t*-test was used for comparison between groups; non-normal measure data were expressed as median and quartile, and Wilcoxon rank-sum test in non-parametric test was used for comparison between groups. A risk prediction model was developed using the binary logistic regression analysis, and the model’s distinction and calibration were evaluated using the receiver operating characteristic (ROC) area under the curve (AUC) and Hosmer–Lemeshow (H-L) goodness-of-fit test. The application efficacy of the model was verified by its accuracy, with a significance level of *p* < 0.05 was used in this study.

## 3 Results

### 3.1 Patient characteristics

**Figure 1:**
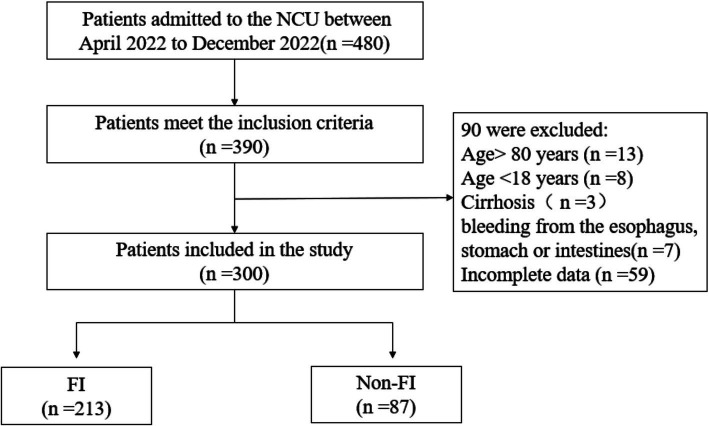
Sampling screening flowchart.

Ultimately, 300 patients were included in this study based on the inclusion and exclusion criteria (Figure 1). Among them, 213 were classified into the FI group, resulting in an overall incidence rate of 71%. Within the FI group, constipation was identified in 105 cases (49.3%), diarrhea in 80 cases (37.6%), reflux/vomiting in 20 cases (9.38%), aspiration in 5 cases (2.34%), high gastric remnants in 2 cases (0.93%), and gastrointestinal bleeding in 1 case (0.5%).

### 3.2 Univariate analysis of FI

The univariate analysis showed statistically significant differences between the two groups in several factors such as age, APACHE II scores, GCS scores, blood glucose, serum albumin, serum lactate, blood potassium, mechanical ventilation, sedation, route of feeding, and temperature ≥ 38.5°C(*p* < 0.05) (Table 1).

**Table 1:** The univariate analysis of the influencing factors of FI (*n* = 300).

Sports event	FI Group (*n* = 213)	Non-FI group (*n* = 87)	*χ^2^*	*p*
Sex [cases (percentage, %)]				
Male	130 (61.03)	57 (65.52)	0.529	0.467
Female	83 (38.97)	30 (34.48)		
Age [years, cases (percentage, %)]				
<60	53 (24.88)	62 (71.26)	56.214	<0.001
≥60	160 (75.12)	25 (28.74)		
BMI [example (percentage, %)]				
≥18.5	203 (95.31)	83 (95.40)	0.001	0.971
<18.5	10 (4.69)	4 (4.60)		
History of alcohol use [cases (percentage, %)]				
Yes	86 (40.38)	33 (37.93)	0.154	0.695
No	127 (59.62)	54 (62.07)		
Smoking history [cases (percentage, %)]				
Yes	87 (40.85)	32 (36.78)	0.426	0.514
No	126 (59.15)	55 (63.22)		
Primary diagnosis [cases (percentage, %)]				
Hypertensive cerebral hemorrhage	69 (32.39)	24 (27.58)	5.812	0.455
Traumatic brain injury	54 (25.35)	17 (19.54)		
Cerebral ischemic stroke	44 (20.66)	28 (32.18)		
Subarachnoid hemorrhage	16 (7.51)	8 (9.20)		
Intracranial tumor	9 (4.23)	3 (3.45)		
Seizures	6 (2.82)	1 (1.15)		
Else	15 (7.04)	6 (6.90)		
Past history [cases (percentage, %)]				
0	104 (34.67)	44 (14.67)	0.002^(1)^	0.964
1	76 (25.33)	28 (9.33)		
2	30 (10.00)	12 (4.00)		
3	2 (0.67)	3(1.00)		
4	1 (0.33)	0 (0.00)		
GCS scores [points, cases (percentage, %)]				
>8	70 (32.86)	59 (67.82)	31.520^(1)^	<0.001
6–8	84 (39.44)	21 (24.14)		
3–5	59 (27.70)	7 (8.05)		
APACHE II scores [points, cases (percentage, %)]				
<20	63 (29.58)	61 (70.11)	41.860	<0.001
≥20	150 (70.42)	26 (29.89)		
NRS 2002 score (points, x ± s)	3.34 ± 0.494	3.36 ± 0.505	0.773^(2)^	0.542
Surgery [cases (percentage, %)]				
Yes	137 (64.32)	54 (62.07)	0.135	0.713
No	76 (35.68)	33 (37.93)		
Mechanical ventilation [cases (percentage, %)]				
Yes	181 (84.98)	39 (44.83)	50.916	<0.001
No	32 (15.02)	48 (55.17)		
Target temperature management [cases (percentage, %)]				
Yes	24 (11.27)	13 (14.90)	0.772	0.380
No	189 (88.73)	74 (85.06)		
Type of antibiotic [cases (percentage, %)]				
≥2	46 (21.60)	20 (22.99)	0.070	0.792
<2	167 (78.40)	67 (77.01)		
Use of vasoactive drugs [cases (percentage, %)]				
Yes	163 (76.53)	59 (67.82)	2.435	0.119
No	50 (23.47)	28 (32.18)		
Use of tranquilizers [cases (percentage, %)]				
Yes	141 (66.20)	43 (49.43)	7.327	0.007
No	72 (33.80)	44 (50.57)		
Use of analgesics [cases (percentage, %)]				
Yes	157 (73.70)	58 (66.67)	1.509	0.219
No	56 (26.29)	29 (33.33)		
Use of gastrointestinal potassium preparations [cases (percentage, %)]				
Yes	195 (91.55)	82 (94.25)	0.638	0.425
No	18 (8.45)	5 (5.75)		
Use of acid suppressants [Example (percentage, %)]				
Yes	24 (11.27)	9 (10.34)	0.054	0.817
No	189 (88.73)	78 (89.66)		
Route of feeding [cases (percentage, %)]				
Nasointestinal tube	17 (7.98)	39 (44.83)	55.237	<0.001
Nasointestinal tube	196 (92.02)	48 (55.17)		
Name of nasal preparation [Example (percentage, %)]				
Standardized Whole Protein Formula	53 (24.88)	21 (24.14)	0.018	0.892
Contains cellulose and a whole protein formula	160 (75.12)	66 (75.86)		
Body temperature ≥ 38.5°C [cases (percentage, %)]				
Yes	121 (56.81)	38 (43.68)	4.275	0.039
No	92 (43.19)	49 (56.32)		
Blood glucose values [mmol/L, example (percentage, %)]				
≤11.1	149 (69.95)	81 (93.10)	18.506	<0.001
>11.1	64 (30.05)	6 (6.90)		
Serum albumin [g/L, example (percentage, %)]				
≥30	110 (51.64)	80 (91.95)	43.223	<0.001
<30	103 (48.36)	7 (8.05)		
Prealbumin [ng/L, example (percentage, %)]				
≥150	142 (66.67)	61 (70.11)	0.336	0.562
<150	71 (3.33)	26 (29.89)		
Serum lactate (min, ^−^χ ± s)	2.21 ± 1.23	1.95 ± 0.83	0.033^(2)^	0.001
Blood potassium stratification [mmol/L, cases (percentage, %)]				
≥3.5	105 (49.30)	30 (34.48)	5.476	0.019
<3.5	108 (50.70)	57 (65.52)		

(1) *z*-value; (2) *t*-value.

### 3.3 Logistic regression analysis of FI

Logistic regression analyses were conducted with the incidence of FI as the dependent variable and the variables with significant differences in the univariate analysis as the independent variables. The assignments of variables are shown in Table 2. Logistic regression analyses showed that seven factors were associated with FI, including age, GCS scores, APACHE II scores, route of feeding, mechanical ventilation, hyperglycemia, and serum albumin. The visualization results of the logistic regression analyses are shown in Table 3.

**Table 2:** Independent variable assignment table.

Variables	Assignment description
Age	<60 = 1, ≥60 = 2
GCS score	>8 = 1, 6–8 = 2, 3–5 = 3
APACHE II score	<20 = 1, ≥20 = 2
Hyperglycemia	≤11.1 = 1, >11.1 = 2
Serum albumin	≥30 = 1, <30 = 2
Tranquilizers	No = 0, Yes = 1
Mechanical ventilation	No = 0, Yes = 1
Route of feeding	nasoenteric tube = 1, nasogastric tube = 2
Temperature status (hyperthermia ≥38.5)	No = 0, Yes = 1
Serum lactate	Substitute the original value
Potassium	≥3.5 = 1, <3.5 = 2

**Table 3:** Logistic regression analysis of risk factors for developing FI in neurocritical care patients.

Independent variable	*B*	*SE*	*Wald*	*p*	*OR (95% CI)*
Constant	−14.373	2.063	48.56	<0.001	0.000
Mechanical ventilation	1.184	0.437	7.327	0.007	3.267 (1.386–7.699)
Route of feeding	2.309	0.515	20.074	<0.001	10.068 (3.666–27.648)
Age	1.65	0.418	15.561	<0.001	5.208 (2.294–11.823)
GCS layering			12.294	0.002	
GCS 6–8 points	1.336	0.484	7.636	0.006	3.805 (1.475–9.817)
GCS 3–5 points	1.696	0.617	7.564	0.006	5.452 (1.628–18.255)
APACHE II score	1.753	0.445	15.486	<0.001	5.771 (2.410–13.816)
Hypoglycemia	1.683	0.630	7.146	0.008	5.383 (1.567–18.491)
Serum albumin	1.954	0.523	13.96	<0.001	7.060 (2.532–19.682)

### 3.4 Development of model

The partial regression coefficients of the FI independent predictors determined based on the binary logistic regression analysis were used to develop the model. The fitted regression equation of the FI risk prediction model for neurocritical patients is as follows: Logit *p* = $\ln\frac{P}{1-p}$ = − 14.737 + 1.184 × mechanical ventilation+2.309 × route of feeding +1.650 × age+1.336 × GCS tertile (6–8 points) + 1.696 × GCS tertile (3–5 points) + 1.753 × APACHE II scores+1.683 × blood glucose value+1.954 × serum albumin.

### 3.5 Validation of model

**Figure 2:**
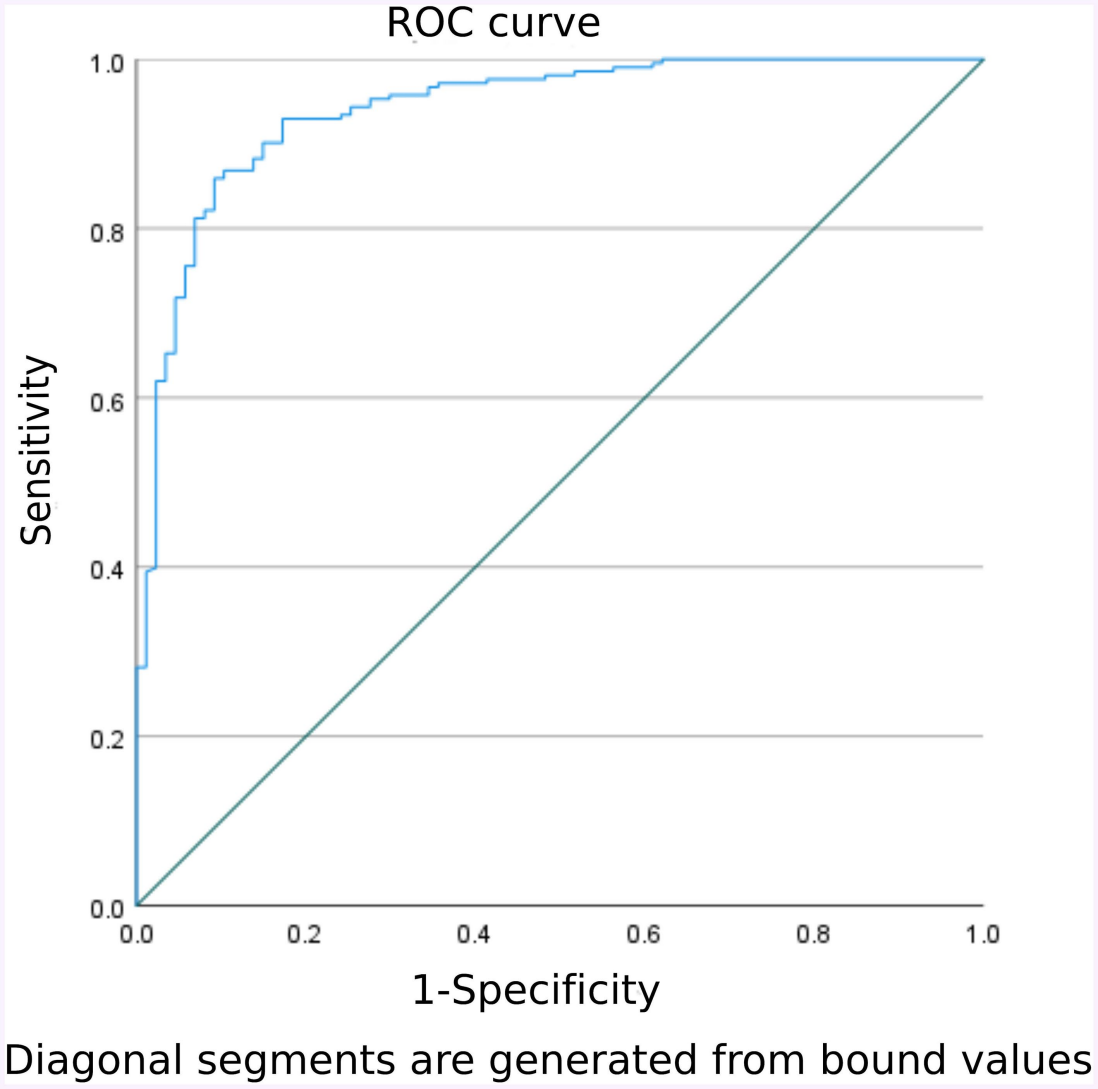
Validation group model predicts ROC curves for early enteral nutrition FI in NCU patients.

The goodness of fit of the model was tested using the H-L test, yielding a *χ^2^* value of 9.622 with *P* = 0.293(> 0.05). This indicates that the predictive ability of the FI risk model closely aligns with the actual incidence rate, demonstrating good compliance. The ROC curve was plotted to calculate the AUC, resulting in an AUC of 0.941 with a 95% CI of 0.912–0.970, *p* < 0.001. When the optimal risk cutoff value was set at 0.767%, the sensitivity of the model was 85.9% and the specificity of the model was 90.8%. At this point, the Youden index reached its maximum at 0.715, signifying that the constructed FI prediction model had a better ability to discriminate whether FI occurs or not. See Figure 2 for further details.

## 4 Discussion

In this study, the incidence of FI was found to be 71%, which is a high rate and aligns with previous research conducted by Pinto et al. (15) and Zhu et al. (12). After brain injuries in NCU patients, under the interactive influence of brain–gut axis, the inhibited vagus nerve weakens the patients’ gastrointestinal motility, which is often manifested by symptoms such as abdominal distension, constipation, and high GRV clinically (35, 36). Simultaneously, under the stress response of the body, there is a heightened risk of acute damage to the intestinal mucosal barrier (37, 38), and this condition facilitates the entry of bacteria, endotoxin, and intestinal cytokines into the bloodstream, further aggravating gastrointestinal dysfunction (39). Additionally, due to insufficient organ perfusion, gastrointestinal edema, and a significant decrease in intestinal absorptive capacity (40, 41), patients become more susceptible to clinical manifestations of FI, such as vomiting and diarrhea. Therefore, from a pathophysiological perspective, NCU patients are more prone to FI compared to other patients. Clinical nurses should pay attention to FI and take proactive measures to reduce its incidence.

The current study revealed that age ≥ 60 years is an independent risk factor for FI during early enteral nutrition in NCU patients, which is in agreement with the findings of Tao et al. (42). It may be related to the pathophysiological mechanism of the human body. As age advances, intestinal function tends to decline and undergo atrophy, accompanied by a reduction in digestive function and absorption capacity. Consequently, this phenomenon increases the risk of FI.

According to the definition associated with the GCS score, when the GCS score is less than 8, the patient is considered to be in a comatose state (i.e., severely impaired in consciousness), of which a score of 3–5 indicates a deep coma, while a score of 6–8 indicates a light coma. The study found that a lower GCS score and a higher APACHE II score are associated with an increased risk of FI, which is consistent with the findings of Li et al. (43) and Yang et al. (44). As early as 2011, Chen et al. (45) pointed out the heightened risk of FI in patients with neurological disorders. Lower GCS scores signal more severe brain injuries, resulting in increased intestinal ischemia and edema (46). Conversely, higher APACHE II scores indicate a greater severity of the patient’s condition. At such levels, the body’s stress response is increased, and the systemic inflammatory response leads to increased protein consumption, intestinal dysbiosis, intestinal mucosal barrier damage, and reduced gastrointestinal tolerance, especially for patients with an APACHE II score of >20. Their monitoring should be strengthened (47), and healthcare providers should also be vigilant regarding the potential occurrence of FI in these patients.

The risk of FI in this study was greater in NCU patients who were mechanically ventilated during early enteral nutrition, which is in line with the results of several studies (21, 48). The findings from studies by Blaser et al. (19) and Reintam et al. (49) indicated that the risk of FI in mechanically ventilated patients was as high as 80.2 to 85%. Mechanical ventilation can impair the function of the lower esophageal sphincter and cause gastroesophageal reflux. The endotracheal tube cuff compresses the esophagus, which can result in pharyngeal muscle-wasting atrophy (49). Mechanical ventilation also increases patients’ intrathoracic and intra-abdominal pressures, particularly with prolonged use of high levels of positive end-expiratory pressure (PEEP). This can lead to a decrease in cardiac output and peripheral organ perfusion, resulting in a reduced blood supply to the gastrointestinal tract, and ultimately increasing the risk of FI. Therefore, it is essential to closely monitor mechanically ventilated patients, especially those who use high levels of PEEP, strengthen the assessment of the patient’s respiratory status, and stop the mechanical ventilation therapy as soon as possible.

This study showed that the route of enteral feeding was also one of the independent risk factors for FI in NCU patients. Patients with neurological impairment were at a high risk of aspiration, often accompanied by increased intracranial pressure, and prone to choking and vomiting (50). For NCU patients, tube feeding is often required due to diminished state of consciousness and the use of mechanical ventilation (1). Guidelines recommend that patients requiring tube feeding should opt for nasogastric tube feeding. For those at a high risk of aspiration or with elevated gastric residual volume, post-pyloric feeding, preferably through the nasoenteric tube, is recommended (51). In 2021, a meta-analysis revealed that (52) post-pyloric feeding can significantly reduce the incidence of gastrointestinal complications such as reflux, vomiting, diarrhea, bloating, gastric remnant, and constipation. Subsequently, in 2022, the Chinese Nursing Association (CNA) issued an expert consensus on enteral nutrition for NCU patients (27). It is recommended that for NCU patients, if the GRV is >100 mL and does not improve after 48 h of adjusting the infusion rate or using gastric prokinetic drugs, the placement of a nasoenteric tube is advised.

The results of this study showed that hyperglycemia before feeding would increase the risk of FI. Hyperglycemia reflexively reduces gastric tone and exacerbates gastric retention, but it also accelerates the pyloric activity, contributing to uncoordinated gastroduodenal contractions and impaired gastric emptying (8), which are also consistent with the results of Nguyen et al.’s study (53). For NCU patients, blood glucose levels are also an important factor affecting disease prognosis (54). According to critical patient blood glucose management guidelines (55), controlling blood glucose levels at approximately 7.8–10.0 mmol/L is relatively safe. However, there are various factors affecting patients’ blood glucose levels. Therefore, personalized blood glucose management according to patients’ different disease conditions, including the selection of medication and enteral nutritional preparations, is essential in clinical practice. It will be the focus of the blood glucose management of NCU patients in the future.

In this study, patients with low levels of serum albumin were also identified as being at risk for FI. Serum albumin serves many vital physiological functions, such as maintaining cellular osmotic pressure, scavenging oxygen-free radicals, and transporting fatty acids and cholesterol (56). When hypoproteinemia occurs, osmotic pressure changes and gastrointestinal mucosal edema increases the incidence of FI (21, 57). Dynamic monitoring in clinical settings should be strengthened, and timely supplementation is necessary when hypoalbuminemia occurs to reduce the incidence of FI.

In this study, the univariate analysis of sedation showed statistical significance. However, the results of the multivariate analysis showed that sedation was not an independent risk factor for FI in patients. Sedative drugs inhibit the release of excitatory neurotransmitters, leading to slower bowel movements. Previous studies (58) have proven that higher doses of sedatives are associated with more severe gastrointestinal dyskinesia. It is possible that there may be a difference between the results derived from unifactorial and multifactorial analyses in the prediction model, and the reasons for this difference could be analyzed in relation to the specific type of drug used, the dose used, and the duration of its use. Future studies can further focus on the effects of the above drugs on patients’ FI. In addition, some predictors, such as specific gastrointestinal biomarkers, or more granular clinical factors (e.g., inflammation markers), were not included in the model in the present study, considering their clinical utility as well as measurement accuracy. These aspects can be further explored in future studies.

The area under the ROC curve of this model was 0.941, with a 95% CI of 0.912–0.970, and the H-L goodness-of-fit resulted in a *p*-value of 0.293 (>0.05), indicating that the model had a good ability to predict whether FI occurred in practice. In clinical application, the area under the ROC curve was 0.924, with a 95% CI of 0.878–0.970 (*p* < 0.001); when the optimal cut-off value was set at 46.3%, the sensitivity of the model was 96.0%, the specificity was 74.4%, and the Youden index was 0.704, indicating that the model facilitates healthcare professionals to efficiently and accurately identify the high-risk group of FI among enteral nutrition patients in NCU. Thus, positive and effective measures can be implemented for this high-risk group. These measures include, but are not limited to, the rational selection of feeding routes for enteral nutrition, the establishment of a scientific, standardized implementation plan for enteral nutrition, the appropriate use of pro-gastric motivational drugs, the use of probiotics, the addition of soluble dietary fiber, early rehabilitation of critically ill patients, and traditional Chinese medicine therapies such as massage. In conclusion, early identification and early intervention are crucial steps in reducing the risk of FI.

## 5 Limitations

This study had some limitations. First, the data used to develop the prediction model were drawn exclusively from patients in a single tertiary hospital, which may limit the representativeness of the sample and suggest that the results require further validation. Second, the study did not explore important clinical outcomes such as ICU length of stay or mortality, which could be areas for future research. Finally, while we conducted internal validation of the model, external validation was not performed. Thus, the predictive effectiveness of the model should be continuously validated in future studies to enable broader generalization.

## 6 Conclusion

The incidence of FI in NCU patients was notably high, with independent risk factors identified as age, GCS scores, APACHE II scores, feeding route, mechanical ventilation, hypoglycemia, and serum albumin levels. Therefore, early screening for FI risk in these patients may be particularly important to reduce its occurrence. This study developed a predictive model for FI risk in NCU patients, demonstrating strong predictive performance and clinical utility. The model may be used as an objective and convenient screening tool for the early identification of high-risk patients, enabling the implementation of appropriate interventions to mitigate FI occurrence and improve patient outcomes.

## Data Availability

The original contributions presented in the study are included in the article/supplementary material, further inquiries can be directed to the corresponding authors.
